# Metabolic Shifts as the Hallmark of Most Common Diseases: The Quest for the Underlying Unity

**DOI:** 10.3390/ijms22083972

**Published:** 2021-04-12

**Authors:** Laurent Schwartz, Marc Henry, Khalid O. Alfarouk, Stephan J. Reshkin, Miroslav Radman

**Affiliations:** 1 Assistance Publique des Hôpitaux de Paris, 75004 Paris, France; 2 Laboratoire de Chimie Moléculaire de l’Etat Solide, UMR 7140 UDS-CNRS, University of Strasbourg, 4 rue Blaise Pascal, F-67000 Strasbourg, France; henry@unistra.fr; 3 Department of Pharmacology, College of Pharmacy, Zamzam University College, Khartoum 11123, Sudan; Alfarouk@Hala-Alfarouk.org; 4 Department of EMS, Al-Ghad International College for Applied Medical Sciences, Al-Madinah Al-Munwarah 42316, Saudi Arabia; 5 Department of Bioscience, Biotechnology and Biopharmaceutics, University of Bari, 70126 Bari, Italy; stephanjoel.reshkin@uniba.it; 6 Mediterranean Institute for Life Sciences (MedILS), 21000 Split, Croatia; miroslav.radman@medils.hr

**Keywords:** Alzheimer, psychiatry, cancer, entropy, pHi, mitochondria, lactic acid, methylene blue, a paradigm shift

## Abstract

A hyper-specialization characterizes modern medicine with the consequence of classifying the various diseases of the body into unrelated categories. Such a broad diversification of medicine goes in the opposite direction of physics, which eagerly looks for unification. We argue that unification should also apply to medicine. In accordance with the second principle of thermodynamics, the cell must release its entropy either in the form of heat (catabolism) or biomass (anabolism). There is a decreased flow of entropy outside the body due to an age-related reduction in mitochondrial entropy yield resulting in increased release of entropy in the form of biomass. This shift toward anabolism has been known in oncology as Warburg-effect. The shift toward anabolism has been reported in most diseases. This quest for a single framework is reinforced by the fact that inflammation (also called the immune response) is involved in nearly every disease. This strongly suggests that despite their apparent disparity, there is an underlying unity in the diseases. This also offers guidelines for the repurposing of old drugs.

## 1. Introduction

From a thermodynamic standpoint, cell viability feeds on low entropy molecules such as glucose to release higher entropy molecules such as CO_2_ and ATP [1]. Low entropy compounds are absorbed by the cells and degraded into higher entropy either in the form of heat (catabolism) or biomass (anabolism), complying with the second law of thermodynamics [2,3,4].

Metabolism is the ensemble of life-sustaining chemical transformations within the cells. Indeed, cell metabolism is not solely the sum of all the chemical reactions and dynamic exchanges between a cell and its microenvironment, but it is primarily the core executing, performing, and operating life continuum. Comparative analyses of genes and genomes from organisms belonging to Eukarya reveal that, during evolution, there have been limited changes, slight evolutionary flexibility in the evolution of cellular metabolism (amino acids, carbohydrates, and lipid metabolism), to support basic functions of life [5].

As an open system, a cell selectively uptakes various compounds from its microenvironment, first deconstructing simple sugars modifying their sub-units along anabolic metabolic pathways, for then building up a set of macromolecules having crucial functions such as DNA, proteins, and enzymes, lipids, etc., enabling to maintain metabolic activity. The capture of free energy from molecular bond rearrangement of carbon sources in catabolic reactions by means of enzymes coupled to energy currency (ATP/ADP) and redox cofactors (NADH/NAD^+^ and NADPH/NADP^+^) powers anabolic reactions that sustain function [4]. These enzyme-catalyzed reactions allow organisms to grow and reproduce, maintain their structures, and respond to their environments.

In most cells, dingle glucose catabolism (e.g., glycolysis pathway) results in two ATP molecules (by recycling two ADP molecules) while converting one mole of glucose to two moles of pyruvate. However, in respiring cells, up to 34 ATP molecules are obtained, and the 2 ATP from glycolysis. Cell respiration is an oxidative phosphorylation (OXPHOS) process. Cells thus convert low entropy glucose by means of an electron-proton transfer process to high entropy ATP from ADP [1]. The energy of electron flow is stored in the form of chemical-free energy of the phosphate-phosphate bond in ATP molecule, which is then used to execute mechanical, osmotic, and biosynthetic work supporting cell functions, viability, and growth [6]. OXPHOS occurs within mitochondria; electrical charges are transferred to oxygen via redox reactions, and protons are pumped from the matrix across the mitochondrial inner membrane. ATP is synthesized when protons return to the mitochondrial matrix down their electrochemical gradient. The rate of entropy production in OXPHOS is determined by the bio-membrane conductance and the electromotive potential across the mitochondrial membrane [4].

## 2. Cells Proliferate under Redox Conditions and Differentiate from Oxidation

The mitochondrion seems to be more than just an efficient power plant for ATP turnover [7,8]. Mitochondria are at the core of eukaryotic cell metabolism and cell differentiation [1]. They also control the release of entropy in the form of heat.

Differentiated cells have an increased mitochondrial activity [7,8,9], resulting in the release of entropy in the form of thermal photons. Maturation of the mitochondrial network, as well as increased transcription of mtDNA, is observed during the differentiation of hESCs into cardiomyocytes [10], in differentiating hESCs [11], in osteogenic adipogenic and hepatogenic differentiation [12], or leukemia cell differentiation [13]. T-cell maturation involves the progression from anaerobic glycolysis to oxidative phosphorylation [14,15]. Transplantation of energy-producing mitochondria results in cell differentiation [16,17].

Differentiated cells have a basal oxidative metabolism. Pyruvate is converted from glucose and degraded by the efficient TCA cycle [18,19]. The oxidative phosphorylation of acetyl-CoA into mitochondria yields large amounts of entropy-rich ATP and releases carbon dioxide and water as waste products.

The opposite occurs in proliferative cells. The carbon flux is rewired to biomass synthesis and cell growth. Glycolysis is then shunted to the pentose phosphate pathway (PPP), generating nucleic acid precursors for DNA replication [18,19,20]. Poorly differentiated cells release their entropy in the form of biomass [1]. Undifferentiated cells have lower mitochondrial activity resulting in alkaline pH, a lower transmembrane potential, and faster cell division [21].

Cells oscillate between two modes of entropy production. Differentiated cells release entropy in the form of heat. They have high ATP production, increased transmembrane potential, increased ionic concentration, intracellular acidic pH, and higher water activity. On the other hand, proliferative cells have decreased ATP synthesis, diluted ionic content, low transmembrane potential, alkaline pH [7]. They release most of their entropy in the form of biomass.

## 3. Metabolic Shifts in a Broad Spectrum of Diseases

Anabolism and catabolism are not on/off phenomena. During adulthood, respiration is predominant [22]. Childhood and aging are more anabolic than adulthood. In childhood, anabolism results mostly in growth. In aging, anabolism results in age-related diseases such as cancer and Alzheimer’s disease.

Cells in early childhood experience a high proliferation rate resulting in cell multiplication and steady growth. Growth lasts up to puberty. Body growth is fast (about 20 cm per year) during early childhood and then slows down. A peak in growth is followed by growth cessation in puberty [23]. Growth stops when the hormones increase muscle strength resulting in increased mechanical pressure on the chondrocytes. Because of increased physical constraints, chondrocytes stop proliferating and differentiate into bone cells [23].

During aging, there is a shift toward anabolism. The reason for the shift toward anabolism is a decrease in mitochondrial function [17]. Age-related impairment in respiratory enzymes decreases ATP synthesis and enhances reactive oxygen species (ROS) production by increased electron leakage in the respiratory chain. When exposed to high ROS, proteins and nucleic acids are susceptible to oxidative damage, leading to an increased mtDNA mutation rate [24]. Aging is also associated with declines in the capacity of various cell types, including neurons, to respond to metabolic stress due to impairment of mitochondrial function [24,25].

This shift toward anabolism was first described by the Nobel Prize winner Otto Warburg (1883–1970) in the 1920s. The Warburg effect is a modified cellular metabolism based on aerobic fermentation, which tends to favor anaerobic glycolysis rather than oxidative phosphorylation, even in the presence of oxygen. The Warburg effect results in the release of lactic acid in the extracellular space, the concomitant activation of the Pentose Phosphate Pathway, and anabolism [20].

The Warburg effect was first described in cancer, where it results in the synthesis of new proliferating cells [26]. The Warburg effect has been described in Alzheimer’s and Parkinson’s diseases [24,27].

Metabolic shifts are not limited to age-related diseases. An example is an anxiety. Catecholamines induce decreased mitochondrial activity resulting in the secretion of lactate [28,29]. In 1967, Pitts and McClure suggested that all anxiety symptoms are caused by a raised blood and body fluids lactate level [30]. Hollander has confirmed their work [31,32]. Since then, Sajdyk demonstrated that the infusion of lactate results in anxiety in rats. Lactate infusion is associated with significant regional blood flow changes in panicking patients but not in the non-panicking patients [33].

The shift toward lactate synthesis has been demonstrated in a large spectrum of psychiatric diseases. There is an increased concentration of lactate [34,35] in the sera of patients with autism spectrum disorders. MRI analysis confirmed the increased concentration of lactate in the brain of autistic patients [36]. There were increased cerebrospinal fluid (CSF) lactate concentrations in patients with bipolar disorder and schizophrenia [37]. In 1956, Altshule showed that abnormally large amounts of lactic acid accumulate in the blood after administering glucose in patients with schizophrenic or manic-depressive psychoses [29], confirming the metabolic shifts. The metabolic shift results from an alteration of complex IV of the mitochondria [38]. In autism, such an alteration of the complex IV has been reported [39]. There is a decreased activity of complex IV in schizophrenia [40] and depression [41].

Lactic acid is both the consequence of the metabolic shift and part of the reason for the diseases. Neurons feed on lactate released by glial cells [27]. The increased secretion of lactate by glial cells results in increased uptake by neurons and intracellular acidosis [27]. A fall in pHi decreases neuronal activity [42]. This is in line with neurons’ exposition to increased lactic acid concentration that results in swelling and apoptosis [43]. The acidic intracellular pH has another significant metabolic consequence: a decreased uptake of glucose [27]. PET scan examination with the [18F]-fluorodeoxyglucose of the brain of psychiatric patients shows a decreased uptake of glucose in the cortex [44].

## 4. Inflammation in a Broad Spectrum of Diseases

Inflammation is part of the complex biological response of body tissues to harmful stimuli, such as pathogens, damaged cells, or irritants. It is a protective response involving immune cells, blood vessels, and molecular mediators. The function of inflammation is to eliminate the initial cause of cell injury, clear out necrotic cells and tissues damaged by the original insult and the inflammatory process, and initiate tissue repair. The four classical signs of inflammation are heat, pain, redness, swelling (calor, dolor, rubor, tumor).

At the onset of an infection, burn, or other injuries, these immune programs undergo activation and release inflammatory mediators responsible for the clinical signs of inflammation. Vasodilation and its resulting increased blood flow cause redness (rubor) and increased heat (calor). Increased permeability of the blood vessels results in a leakage of plasma proteins and edema, which manifests itself as swelling (tumor). The mediator molecules also alter the blood vessels to permit the extravasation of leukocytes into the tissue.

There is an inflammatory component in most, if not all, chronic diseases (Table 1). In autism spectrum disorder (ASD), postmortem microscopic analysis points toward an inflammatory disease linked to the blood-brain barrier’s disruption [45]. There is also evidence of inflammation close to the neuron and the microglia with mast cells’ proliferation [46]. Analysis of the brain tissue confirms the inflammation with increased secretion of multiple cytokines and lymphokines (TNF-α, IL-6, GM-CSF, IFN-γ, and IL-8) [47]. This inflammatory syndrome can have various causes or risk factors such as genetics, infections, toxins, fetal restriction, and auto-immune diseases. ASD can be associated with a specific syndrome [48], such as Rett Syndrome, Fragile X syndrome, or 22q13 deletion. Rett syndrome is a neurodevelopmental disorder, which presents itself with neurologic defects. It is most frequently transmitted as an X-linked dominant disease linked to new methyl-CpG-binding protein gene mutations (MECP2). There is evidence of inflammation and dysregulation of the immune system early in life in this syndrome [45]. Fragile X syndrome is associated with brain inflammation [49] with impaired immune response and over-reactive astrocytes. The 22q13 deletion is a genetic disorder caused by the deletion or disruption of segment 13 of the long arm of chromosome 22 [50]. There are multiple neurological features such as hypotonia, delayed speech, or autistic behavior. There are concomitant brain inflammation and auto-immune disease. Treatment with valproic acid is a well-recognized model of autism. Animals treated with prenatal valproic acid have reduced social interaction, decreased exploratory activity, and decreased prefrontal cortex mitochondrial complex activity. They show brain inflammation, oxidative stress, and increased blood-brain barrier permeability [51].

Inflammation has been described in every psychiatric disorder. Eugen Bleuler remarked in 1911: «The fragility of the blood vessels which appears in many schizophrenics, both acute and chronic, seems to indicate a real vascular pathology.» An association between inflammatory abnormalities and schizophrenia has been found repeatedly [52]. Van Kesteren analyzed the brain of the patients who died of schizophrenia and found a consistent brain inflammation report [53]. Similarly to ASD, there is in schizophrenia an inflammatory syndrome with increased secretion of interleukin (IL) 1-beta, IL-6, and transforming growth factor-beta [52]. Brain inflammation has been described in bipolar disease [54], depression [55], Nieman Pick [56], or anorexia nervosa [57].

**Table 1:** Every disease has an inflammatory component associated with malfunctioning mitochondria and increased secretion of lactic acid resulting from metabolic rewiring.

Organ	Disease	Inflammation	Mitochondrial Impairment	Lactic Acid Concentration
Brain	Autism	Yes	Yes [58]	Increased [59]
	Schizophrenia	Yes	Yes [58]	Increased [60]
	Meningitis	Yes	Yes [61]	Increased [60]
	Encephalitis	Yes	Yes [62]	Increased [63]
	Alzheimer	Yes	Yes [27]	Increased [27]
	Parkinson	Yes	Yes [64]	Stable under treatment [65]
	Huntington	Yes	Yes [66]	Increased [67]
Cardio-vascular	Cardiac infract	Yes: scarring	Yes [68]	Increased [69]
	Cardiac failure	Yes	Yes [70]	Increased [69]
	Stroke	Yes: scarring	Yes [71]	Increased [72]
Bronchia alveolar	Infection	Yes	Yes [73]	Not available
	Fibrosis	Yes	Yes [74]	Increased [75]
	Emphysema	Yes	Yes [76]	Increased [77]
	Cancer	Yes	Yes [27]	Increased [27]
Joint and muscular	Arthritis	Yes	Yes [78]	Increased [77]
	Myositis	Yes	Yes [79]	Not available
	Sarcoma	Yes	Yes [27]	Increased [27]
GI tract	Hepatitis	Yes	Yes [80]	Increased [80]
	Cirrhosis	Yes	Yes [81]	Increased [82]
	ulcerative colitis	Yes	Yes [83]	Increased [84]
Urinary tract	Cystitis	Yes	Yes [85]	Increased [86]
	Cancer	Yes	Yes [27]	Increased [27]
Autoimmune disease	Scleroderma	Yes	Yes [87]	Not available
	Lupus	Yes	Yes [88]	Increased [89]
	Sarcoidosis	Yes	Yes [90]	Increased [91]
Down’ syndrome	Diffuse	Yes	Yes [92]	Increased [93]
Cystic fibrosis	Lung and GI tract	Yes	Yes [94]	Increased [95]
Aging	Diffuse	Yes	Yes [27]	Increased [27]

## 5. Inflammation Is Responsible for Metabolic Shifts

Inflammation (a clinical feature) is closely related to hyperosmolarity (a physical characteristic) [96,97]. Animal models of inflammation demonstrate that, in an inflammatory fluid, whatever its cause, there is an increased protein content resulting in increased osmolarity (oncotic pressure). On the other hand, increased osmolarity, whatever its cause, results in inflammation [97]. Therefore, it is positive feedback control. Increased extracellular osmolarity increases cytokine synthesis and secretion and results in the proliferation and activation of immune cells [96]. Several reports claim hyperosmotic contents in the feces of patients suffering from inflammatory bowel disease [96,98,99]. GI tract lesions are caused by increased osmolarity.

An example is dextran sulfate sodium (DSS) induced colitis [100,101]. DSS is chemically inert. It is the hyperosmolarity caused by DSS, which causes colitis. When DSS is ingested at an osmolarity lower than 300 mosmol/L, it displays no toxicity. At higher osmolarity, DSS induces dose-dependent colitis [102]. When the mouse is exposed to DSS, the chemical stays in the GI tract, but the distant lymph node is enlarged with a proliferation of the lymphocytes secondary to the extracellular space’s widespread hyperosmolarity [102].

Hyperosmolarity has a dual effect. It can both stimulate the metabolism and induce apoptosis of cells. Hyperosmolarity induces the secretion of neurotransmitters [103]. In rodents, porcine and human loss of blood-brain barrier integrity by intra-arterial hyperosmotic mannitol has been shown to lead to EEG changes consistent with epileptic seizures, that is, spike/wave complexes interspersed with decreased EEG voltage [104]. Increased pressure exerted by mannitol decreased the amplitude of evoked field potentials and excitatory postsynaptic potentials [105].

There is an inflammatory component in every disease (see Table 2). There is a concomitant rewiring of the metabolic fluxes with an increase secretion of lactic acid. The increased pressure, such as inflammation, inhibits the mitochondria and induces lactic acid secretion [38]. The increased secretion of lactic acid, a stigma of the metabolic shift toward anabolism, feeds on the inflammatory cells and plays a part in the immune response, such as seen in all these diseases (see Table 2). This is in line with the concomitant finding of inflammation, mitochondrial impairment, and lactic acid secretion in most chronic diseases. Intraperitoneal injections in rats of hypertonic solutions result in the secretion of lactate by the brain cells [106].

One way to interpret the effect of inflammation on the activation of the onset of latent diseases (e.g., cancer) is that inflammation interrupts the phenotypic suppression of initiated diseases by interrupting cellular parabiosis; cellular parabiosis is trans-cellular complementation of recessive cell defects by the healthy neighboring cells via intercellular molecular traffic through tube-like connections between cells [107]. Inflammation-induced matrix metalloproteinases are known to destroy such connections. Cellular parabiosis is key to tissue homeostasis that involves compensation for loss-of-function and averaging cell activities in tissues.

## 6. Intracellular pH and the Consequence of the Metabolic Shifts

There is a shift in mitochondrial activity in almost every disease resulting in increased lactate concentration [108]. In epithelial cells, the Warburg-effect results in cancer [26,108]. It is a longstanding debate whether cancer is one disease or a set of remarkably diverse diseases. For most researchers, various diseases with different prognoses, sites of origin, patterns of spread, and kinetics seem to be linked with cancer. However, despite this apparent complexity, there is underlying unity [96].

The Warburg effect is a bottleneck. The cells cannot burn the glucose because the pyruvate cannot de degraded in the Krebs’ cycle. Evidence of the Warburg’s central role comes when the researcher injects into cancer cells, with a micropipette, normal mitochondria. The growth will stop. These cells have become benign. The injection of the nuclei of cancer cells into normal cells does not increase growth. These cells can still burn glucose because the mitochondria are normal and do not form tumors [26].

The inhibition of the oxidative phosphorylation results in the activation of the anabolic pathway, such as the pentose phosphate pathway necessary for DNA and RNA synthesis [20,109]. The decreased mitochondrial activity has a second consequence: cytoplasm alkalinization because of decreased CO_2_ secretion [18]. Dysregulated pH is emerging as another hallmark of cancer because tumors show a ‘reversed’ pH gradient with a constitutively increased intracellular pH higher than the extracellular pH [110,111,112,113]. This gradient enables cancer progression by promoting proliferation, the evasion of apoptosis, metabolic adaptation, migration, and invasion [111,114,115,116]. In normal cells, the intracellular pH oscillates during the cell cycle between 6.8 and 7.3 [117]. The oscillation of the pH during the cell cycle matches the value of the decompaction of the histones, the RNA polymerase activation, the DNA polymerase activation, and the DNA compaction before mitosis. Carbon dioxide reacts with water to create carbonic acid. Cell transformation or enhanced cancer cell division and chemotherapy resistance are associated with a more alkaline pH [118,119].

The brain has the highest energy consumption of the body (around 20% of the body oxygen and 25% of the glucose) while representing 3% of our body’s mass. Neurons feed on lactate released by glial cells [27]. The increased secretion of lactate by glial cells results in increased uptake by neurons and intracellular acidosis [27]. To perform their normal physiological functions, cells must maintain the intracellular pH (pHi) within the physiological range. Intracellular enzyme activity, cytoskeleton component integration, and cellular growth and differentiation rates are strongly associated with the pHi [21]. Acidic intracellular pHi of the neuron results from the excessive secretion of lactic acid by the surrounding glial cells and results in apoptosis.

In cancer, mitochondrial impairment results in cell proliferation and tumor growth. In Alzheimer’s disease, there is abnormal secretion of amyloid plaques, in Parkinson disease, there are intracellular deposits (Lewy bodies). In cancer, the alkaline pH results in cell proliferation. In neurodegenerative diseases, the acidic pH results in apoptosis [27].

Seen from a biologist’s perspective, most metabolic pathways appear to be connected. However, from a physicist’s standpoint, they all point towards an increased entropy flux within the body. Whatever the cause (i.e., genetic defect within the respiratory chain, inflammation, or toxicity of xenobiotics), they all converge toward a shift in the type of entropy that is produced. In other words, all these diseases have in common a decreased activity of the mitochondria. The synthesis of thermal photons is decreased, and there is a concomitant increase in biomass synthesis. This imbalance can be addressed in treating the primary cause (for example, a genetic defect in the electron transport chain) and/or by a medication targeting the mitochondria such as Methylene Blue. It is of utmost importance to better analyze the patients’ metabolism to target therapy to restore the entropy imbalance.

## 7. Conclusions: Handling the Complexity of Phenotypes in a Single Frame

Up to now, most biological research has focused on isolated single biological reactions. Cell biology became a descriptive detailed molecular approach to “how” without knowing “what” and “why”. It is a science lacking key concepts. Most biomedical research deals with “biomarkers,” which are arbitrary downstream consequences of the cause of disease and the damage done by the disease. Acting upon such biomarkers cannot and does not cure diseases.

However, living organisms are energy-driven intricate integrated systems that should be described as open thermodynamic systems. When understood as physical systems, such organisms appear as open, non-equilibrium thermodynamic open systems exhibiting a hierarchical organization. Therefore, if such systems are to be understood, each component, such as a cell, organelle, or organ, should be subject to a thermodynamic description. To advance our understanding of the biological processes, they need to be evaluated and integrated into comprehensive fundamental theories based on physics principles.

The proposed entropy-centric paradigm for human diseases is placed in the relationship with basic aspects of cellular energy metabolism (Table 2).

## Figures and Tables

**Table 2:** Energy metabolism and entropy in key biological processes.

Entropy Released as Biomass	Entropy Released as Heat
Anabolism	Catabolism
Lactic Fermentation	Respiration
Anaerobic Glycolysis	Oxidative Phosphorylation
Proliferation	Cell Differentiation
Reduction	Oxidation
Low ATP synthesis	High ATP synthesis
Low water activity	High water activity
